# Correction to: COVID-19 and air pollution in Vienna—a time series approach

**DOI:** 10.1007/s00508-021-01901-3

**Published:** 2021-06-17

**Authors:** Hanns Moshammer, Michael Poteser, Hans-Peter Hutter

**Affiliations:** 1 Department of Environmental Health, Center for Public Health, Medical University Vienna, Kinderspitalgasse 15, 1090 Vienna, Austria; 2 Department of Hygiene, Medical University of Karakalpakstan, 230100 Nukus, Uzbekistan

**Correction to:**

**Wien Klin Wochenschr 2021**

10.1007/s00508-021-01881-4

The original version of this article unfortunately contained a mistake in Fig. 2. The correct figure can be found below. The authors apologize for the mistake. The original article has been corrected.

**Fig. 2:**
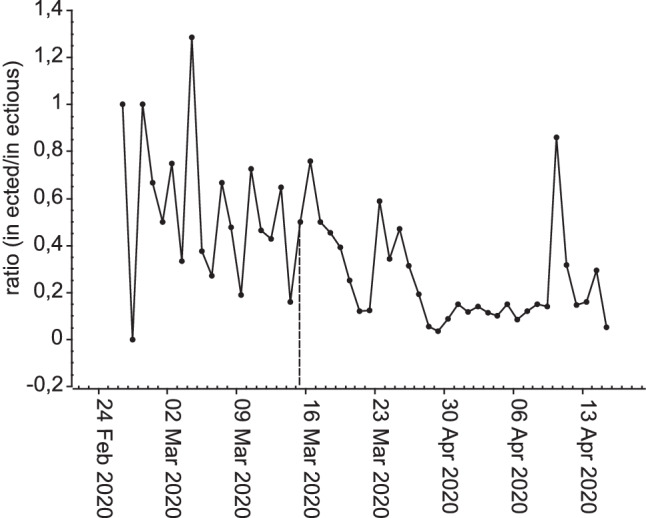
Time course of the estimated ratio of infected by infectious persons in Vienna (23 February 2020 - 21 April 2020) assuming 6 infectious days in total beginning 1 day before diagnosis (March 16th, the onset of measures, marked by a dashed line)

